# Anticonvulsant Medications in Mitochondrial Disease

**DOI:** 10.15844/pedneurbriefs-31-3-3

**Published:** 2017-11

**Authors:** Divakar S. Mithal, Jonathan E. Kurz

**Affiliations:** 1 Division of Neurology, Ann & Robert H. Lurie Children’s Hospital of Chicago, Chicago, IL; and Departments of Pediatrics and Neurology, Northwestern University Feinberg School of Medicine, Chicago, IL

**Keywords:** Mitochondrial Disease, Epilepsy, Anti-Convulsant, Anti-Epileptic Drug

## Abstract

Researchers from Vienna, Austria and Sao Paulo, Brazil studied the known effects of anticonvulsant drugs on mitochondria, using a literature search to include only references to epilepsy associated with mitochondrial disease, and a specific anti-convulsant drug (i.e. levetiracetam) with a specific mitochondrial function (i.e. mitochondrial membrane potential).

Researchers from Vienna, Austria and Sao Paulo, Brazil studied the known effects of anticonvulsant drugs on mitochondria, using a literature search to include only references to epilepsy associated with mitochondrial disease, and a specific anti-convulsant drug (i.e. levetiracetam) with a specific mitochondrial function (i.e. mitochondrial membrane potential). Across 31 different anti-convulsant medications, the authors searched for six mitochondrial functions that anticonvulsant drugs may influence. Some medications, such as valproic acid, were extensively studied across all functions while others, such as rufinamide and stiripentol, had no publications matching the search criteria. For each aspect of mitochondrial biology, the anticonvulsants with either greatest benefit or most toxicity were discussed in greater detail. Subsequently, the overall toxicity and tolerability was assessed. Using these findings, the authors describe an approach to treating mitochondrial epilepsy with drugs featuring the most efficacy and lowest toxicity first, such as lamotrigine, levetiracetam and lacosamide. They suggested using caution with drugs (such as valproic acid and phenytoin) where toxicities outweighed protective effects on mitochondria, although these drugs may remain useful in selected patients. They further recommended exercising caution with intravenous and combination therapies. Finally, they suggested attempting to tailor anticonvulsants to the specific mitochondrial disease diagnosis. Cumulatively, they used the literature search to provide a broad framework for approaching mitochondrial epilepsy. [1]

COMMENTARY. The authors provide a comprehensive overview of the current knowledge regarding mitochondrial effects of anticonvulsants. The breadth of information is a valuable resource; however, the available data are limited and sometimes difficult to interpret.

To date, most experts emphasize avoidance of medications with toxic potential to mitochondria [2, 3]. One well-accepted principle is to avoid valproic acid, particularly for *POLG-1* related disorders. This practice derives from extensive laboratory data [1] coupled with specific clinical reports, such as that of two patients with Alpers-Huttenlocher Syndrome and refractory epilepsy who suffered fulminant liver failure from valproate administration [4]. With the rapid increase in available anticonvulsant therapies and relative infrequency of mitochondrial epilepsy, there is scant evidence for or against other medications. Investigating the exact pharmacologic mechanisms and their effect on mitochondrial function is difficult but remains important.

Although not reviewed in detail, the authors allude to the use of a ketogenic diet (KD) for mitochondrial epilepsy. This has been controversial due to concern over stress induction through KD [5]. KD may provide alternative fuels for damaged mitochondria [3], and recent studies indicate it is safe for mitochondrial epilepsy [6].

Treatment of epilepsy in patients with mitochondrial disease should adhere to best practices for epilepsy therapy with added awareness of the potential for mitochondrial toxicity. The authors effectively use a literature search method to identify common first line treatments [1]. While large studies are still lacking, a consensus is emerging including the use of KD [3, 6]. Data regarding anticonvulsant use in mitochondrial epilepsies remains scarce, underscoring the need for both literature reviews such as this one and large multi-center studies.
